# Functions of Cytochrome *c* Oxidase Assembly Factors

**DOI:** 10.3390/ijms21197254

**Published:** 2020-09-30

**Authors:** Shane A. Watson, Gavin P. McStay

**Affiliations:** Department of Biological Sciences, Faculty of School of Life Sciences and Education, Staffordshire University, Science Centre, Leek Road, Stoke-on-Trent ST4 2DF, UK; w032424h@student.staffs.ac.uk

**Keywords:** mitochondria, cytochrome *c* oxidase, electron transport chain, oxidative phosphorylation

## Abstract

Cytochrome *c* oxidase is the terminal complex of eukaryotic oxidative phosphorylation in mitochondria. This process couples the reduction of electron carriers during metabolism to the reduction of molecular oxygen to water and translocation of protons from the internal mitochondrial matrix to the inter-membrane space. The electrochemical gradient formed is used to generate chemical energy in the form of adenosine triphosphate to power vital cellular processes. Cytochrome *c* oxidase and most oxidative phosphorylation complexes are the product of the nuclear and mitochondrial genomes. This poses a series of topological and temporal steps that must be completed to ensure efficient assembly of the functional enzyme. Many assembly factors have evolved to perform these steps for insertion of protein into the inner mitochondrial membrane, maturation of the polypeptide, incorporation of co-factors and prosthetic groups and to regulate this process. Much of the information about each of these assembly factors has been gleaned from use of the single cell eukaryote *Saccharomyces cerevisiae* and also mutations responsible for human disease. This review will focus on the assembly factors of cytochrome *c* oxidase to highlight some of the outstanding questions in the assembly of this vital enzyme complex.

## 1. Introduction

Energy generation is a fundamental process that supports all forms of life on earth. The universal energy currency in life is adenosine triphosphate (ATP) which captures energy from bioenergetic and metabolic processes powered by substrate-level and oxidative phosphorylation. In eukaryotes a major portion of ATP is generated through mitochondria, a double-membrane organelle derived from a symbiotic relationship at the origin of multicellular life [1]. Mitochondria harbour a series of multi-subunit complexes that perform electron transfer and proton translocation from the internal mitochondrial matrix space to the inter-membrane space (IMS) through the inner mitochondrial (IMM). This generates an electrochemical gradient across the IMM where proton concentration is higher in the IMS than the matrix. The accumulated protons can re-enter the matrix through ATP synthase—a proton transporter that couples the proton gradient to the synthesis of ATP. The electron transport complexes are an ordered series of multi-subunit complexes that accept electrons from carriers such as nicotinamide adenine dinucleotide (NAD^+^/NADH) and flavin adenine dinucleotide (FAD/FADH_2_) produced through metabolic pathways such as glycolysis, citric acid cycle and β-oxidation. NADH is oxidised by NADH dehydrogenase enzymes, such as multi-subunit Complex I in many eukaryotes or single subunit Ndi1p in *Saccharomyces cerevisiae.* The first step in Complex I sees the electrons passing through a series of co-factors and prosthetic groups such as iron-sulphur (Fe-S) clusters and flavin mononucleotide (FMN). The electrons are passed to the membrane soluble lipid based redox carrier co-enzyme Q (ubiquinone/ubiquinol). In addition, FAD/FADH_2_ is reduced by succinate dehydrogenase of the citric acid cycle which also acts as complex II of the electron transport chain (ETC). Complex II contains Fe-S clusters and haem prosthetic groups to again pass electrons to Co-enzyme Q. Reduced Co-enzyme Q (ubiquinol) is oxidised by complex III (co-enzyme Q:cytochrome *c* oxidoreductase) using Fe-S and haem groups to reduce cytochrome *c*, the next mobile electron carrier in the ETC. Cytochrome *c* localises to the IMS face of the IMM to shuttle electrons from complex III to complex IV using a haem prosthetic group. Complex IV (cytochrome *c* oxidase) is the final complex of the ETC catalysing the reduction of molecular oxygen, the terminal electron acceptor to form water via copper and haem groups. The process of electron transfer is coupled to proton translocation from the matrix to the IMS and is mediated by complexes I, III and IV to generate the electrochemical gradient. 

The ETC complexes are multi-subunit assemblies with protein subunits derived from both nuclear and mitochondrial gene products. To ensure efficient assembly of complexes co-ordinated post-translational processing and protein-protein interactions are required. This is accomplished by a stepwise biogenesis pathway that requires the function of specific chaperones. As oxidative phosphorylation (OXPHOS) is an evolutionarily conserved process in all eukaryotes much understanding of the assembly process can be gleaned from the study from all eukaryotic organisms where mitochondria exist. This is true for single cell eukaryotes such as the budding yeast *Saccharomyces cerevisiae* and photosynthetic alga *Chlamydomoas reinhardtii* to multicellular higher eukaryotes such as human and mouse. The combination of genetic manipulation of eukaryotic model organisms and naturally occurring mutations in human disease has provided much information we have at the moment about how assembly of the ETC occurs. The molecular details of the most studied mammalian organisms and yeast OXPHOS complex structure and assembly have very many similarities but also some differences. These differences are in the number of structural subunits forming complexes as well as the post-translational processing required for correct assembly. Furthermore, multicellular eukaryotes express tissue-specific subunits that provide unique functions to the complexes. Budding yeast has been a powerful model system to understand assembly of OXPHOS complexes as they are facultative anaerobes, meaning they can sustain energy requirements in the absence of functional OXPHOS if grown on a carbon source, such as glucose, that does not require the functions of OXPHOS. When grown on carbon sources such as ethanol, glycerol and lactate, that require functional OXPHOS, any defects in assembly or function of the ETC or OXPHOS results in a lack of growth. This has enabled a large number of genes to be identified that are responsible for ETC and OXPHOS assembly and function through powerful genetic and phenotypic analysis [2]. Recently, more homologues have been identified in higher eukaryotes demonstrating the conservation of the assembly of the complex as well as the enzymatic function [3]. 

## 2. Cytochrome *c* Oxidase Structure and Function

In order to understand the assembly pathway of multi-subunit complexes it is important to put this in the context of the fully assembled and functional complex. Each subunit has a defined three-dimensional organisation and interacts with certain subunits, both required for full function. Mammalian and yeast cytochrome *c* oxidase (COX) are composed of 3 subunits derived from mitochondrial DNA and between 8 and 11 subunits derived from nuclear genes. The mitochondrial encoded subunits Cox1p and Cox2p contain prosthetic groups and co-factors required for electron transfer including haem groups in Cox1p and copper ions in Cox1p and Cox2p. These two subunits are also responsible for the translocation of protons from the matrix to the IMS through hydrophilic channels. The post-translational associations of prosthetic groups and co-factors as well as proteolytic processing and membrane insertion are accomplished by a number of chaperones, some specific to COX and others shared with other OXPHOS complexes. All of these chaperones are encoded by the nuclear genome and for the most part functional homologs are conserved from budding yeast to higher mammals with some exceptions. The functions of many of these chaperones have been determined and are conserved; however, there are still some genes without assigned functions or functional homologs [4]. Determination of gene function and functional homologs will allow for a more complete understanding of the process of COX biogenesis.

Defects in OXPHOS are responsible for an array of genetic disorders that impact on tissues and organs with high metabolic demands or dependency on mitochondrial metabolic pathways. Specifically for COX there are a number of mutations in assembly factor and structural genes that cause forms of Leigh Syndrome, mitochondrial Complex IV deficiency and rare syndromes that result in neurological disorders along with impacts on the heart [5], liver [6], kidney [7] and digestive system [8]. The neurological disorders differ in their onset severity depending on the nature of the mutation. For example, encephalopathies display clinical features due to specific mutations in all OXPHOS complexes as well as other mitochondrial genes involved in mitochondrial DNA maintenance, e.g., DNA polymerase γ [9]. Variation in severity is also observed by certain combinations of clinical features, for example, encephalopathies presenting with tubulopathy and hepatopathy, caused by mutations in the Complex III gene BCS1L [10]. Specific mutations associated with these diseases are individually rare, but when combined with other diseases that cause defects in OXPHOS occur at approximately 1 in 5000 births, representing one of the highest incidences of inherited metabolic diseases affecting humans. Through recent advances in diagnosis of mitochondrial disease and investigation of the molecular basis of the disease insights into the functions of assembly factors is improving [11]. 

In humans and other higher mammals, most assembly factors and structural subunits have an orthologue in budding yeast. The human COX enzyme is composed of 14 subunits, which is 3 more than budding yeast. As with most of the nuclear encoded structural subunits these are single trans-membrane spanning polypeptides that surround the core catalytic subunits and do not interfere with conserved interfaces and interactions [12]. Recently, other subunits have been identified as associated with purified COX including the hypoxia inducible gene Rcf1p/HIGD2A which acts as a link between Complex III to support supercomplex formation especially under anaerobic conditions when Cox5bp is expressed [13,14]. The structure of mammalian COX has been known since the 1980s and recently the 14th subunit was identified. NDUFA4 was originally attributed to Complex I, but biochemical and genetic studies revealed deficiencies in COX activity and an equal stoichiometry with other COX structural subunits without impact on Complex I activity [15,16,17]. NDUFA4 has been identified in purified COX containing supercomplex structures determined by X-ray crystallography at the interface of COX dimers [18]. This is further supported by the presence of a homologue in budding yeast, MIN8/YPR010C-A, identified through proteomics [19]. *S. cerevisiae* do not express a multi-subunit Complex I NADH dehydrogenase, fully supporting another role for this gene. It is annotated as a mitochondrial gene of unknown function. Further work is required to understand the function of this gene as this has not been identified as a gene required for respiratory deficiency in previous studies. The function of these extra subunits is subject to speculation. *S. cerevisiae* COX can retain function in the absence of the Cox8p and Cox13p subunits indicating that not all structural subunits are required for activity [20]. COX8A is the human homologue of yeast Cox8p, and it is the smallest subunit of human COX. In contrast to *S.cerevisiae,* a mutation in this subunit is responsible for neurological disorder due to loss of COX function [21]. *S. cerevisiae* COX13 has two homologues in humans-COX6A1 and COX6A2-tissue specific isoforms in the liver and striated muscle, respectively. Mutations in the isoform COX6A1 have been shown to be the cause of Charcot-Marie tooth disease, a neuropathological disorder [22,23]. Mutations in the second isoform COX6A2 can lead to muscle specific Complex IV deficiency [24]. These discrepancies indicate differences in the essential nature of these peripheral subunits. The 3 human specific subunits in human COX are COX6C, COX7C and NDUFA4. Mutations in NDUFA4 are associated with Leigh Disease [25], while mutations in the two other subunits COX6C and COX7C have not been associated with disease. Even though there are differences between the phenotypes of yeast and humans, this should be taken with some caution as some homologous subunits do not have the same common origin [26]. 

## 3. Cytochrome *c* Oxidase Assembly Factors

Biogenesis of multi-subunit complexes requires specific maturation steps for subunits to ensure correct localisation and topology, post-translational modifications and interactions with other subunits. These biogenesis pathways also require quality control points to verify each step has been correctly performed but also to determine whether downstream steps can occur. Assembly factors for COX can be broadly grouped by function including transcription and translational regulation, membrane insertion, proteolysis, co-factor and prosthetic association or an as yet undefined function. The process of COX assembly ensures appropriate association of correctly matured subunits with incorporated co-factors and prosthetic groups when in adequate abundance. This prevents accumulation of mid-assembly intermediates that can cause potentially detrimental effects due to excess reactive oxygen species (ROS) or overloading of protein quality control systems. Due to the number of subunits that need to associate in the IMM, and the number of maturation steps required, there are many assembly factors to ensure each of these steps are performed correctly. The assembly pathway still needs further elucidation to assign functions to specific assembly factors and how certain assembly intermediates interact, but general functions are beginning to be understood (Table 1). 

A stepwise assembly pathway of the Cox1p, Cox2p and Cox3p assembly modules in budding yeast has been determined. Several assembly intermediates and the kinetic relationship of Cox1, Cox2 and Cox3 were identified using metabolic labelling to follow newly synthesized mitochondrial gene products [2,3,4,5,6].

Cox1p is a 12 trans-membrane domain integral membrane protein localised to the IMM. COX1 mRNA is translated by ribosomes that are associated with the membrane insertase Oxa1p with the assistance of Mba1p. Oxa1p is the insertase responsible for the insertion of Cox2p and Cox3p into the IMM also, as well as subunits for other OXPHOS complexes. Cox1p amino and carboxy termini are both located in the IMS indicating a co-translational insertion process. The control of Cox1p IMM insertion has not been studied specifically but trans-membrane domains are typically comprised of aliphatic and aromatic amino acids. Cox1p can be inserted into the IMM in a temperature sensitive OXA1 mutant-indicating an alternative or less efficient insertion process may exist [7]. Cox3p is also inserted via an Oxa1p dependent mechanism, and much like Cox1p can also be inserted when OXA1 function is lost. Cox2p insertion is entirely dependent on Oxa1p as well as the associated proteins Pnt1p, Mss2p and Cox18p [8,9]. Cox2p also requires processing through a Cox20p chaperone dependent mechanism involving the inner membrane peptidase complex (composed of Imp1/2p and Som1p) which removes the amino-terminal 15 amino acids after insertion of the amino-terminus [10]. Once inserted into the membrane these core subunits must associate with assembly factors and structural subunits to form assembly modules, incorporate co-factors and prosthetic groups, that then associate to form the assembled and functional COX enzyme. This process is far from understood but in budding yeast it has been interrogated using pulse-chase labelling and pull-down assays of these assembly factors and structural subunits. This has provided a kinetic pathway of assembly intermediate formation where specific assembly factors and structural subunits associate with each of the core mitochondrial subunits. 

### 3.1. Cytochrome c Oxidase Subunit 1 Assembly

A stepwise assembly pathway of the Cox1p, Cox2p and Cox3p assembly modules in budding yeast where the order of chaperone and structural subunit association with the mitochondrial gene products was determined. Several assembly intermediates and the kinetic relationship of Cox1, Cox2 and Cox3 were identified using metabolic labelling and affinity purification to follow newly synthesized mitochondrial gene products [20,27,28,29,30]. 

Cox1p is a 12 trans-membrane domain integral membrane protein localised to the IMM. COX1 mRNA is translated by ribosomes that are associated with the membrane insertase Oxa1p that is also responsible for the insertion of Cox2p and Cox3p and other OXPHOS complexes in the IMM [31,32,33,34]. Cox1p amino and carboxy termini are both located in the IMS indicating a co-translational insertion process. The control of Cox1p IMM insertion has not been studied specifically but trans-membrane domains are typically comprised of hydrophobic aliphatic and aromatic amino acids. Cox1p can be inserted into the IMM in a temperature sensitive OXA1 mutant-indicating an alternative or less efficient insertion process may exist [33]. Cox3p is also inserted via an Oxa1p dependent mechanism, and much like Cox1p can also be inserted when OXA1 function is lost. Cox2p insertion is entirely dependent on Oxa1p as well as the associated proteins Pnt1p, Mba1p, Mss2p and Cox18p [35,36,37]. Cox2p also requires processing through a Cox20p chaperone dependent mechanism involving the inner membrane peptidase complex (composed of Imp1/2p and Som1p) which removes the amino-terminal 15 amino acids after insertion of the amino-terminus [38]. Once inserted into the membrane these core subunits must associate with assembly factors and structural subunits to form assembly modules, incorporate co-factors and prosthetic groups, that then associate to form the assembled and functional COX enzyme. 

The mammalian process of COX core subunit maturation tends to follow a similar process to that of yeast - but with some subtle differences. Mammalian Oxa1l has been shown to have slightly different activity towards mitochondrial encoded COX core subunits. Early experiments using RNA interference of Oxa1l in HEK293T cells reduced expression of ATP synthase and Complex I subunits with minimal impact on COX subunit expression or activity [32]. More recently, a patient with a single point mutation in OXA1L displayed defects in ATP synthase and COX expression and function that was also observed in *Drosophila melanogaster* and human osteosarcoma U2OS cells, perhaps indicating tissue specific differences for protein insertion in the IMM. OXA1L was also found to associate with Cox1, Cox2 and Cox3 using proteomic analysis of OXA1L immunoprecipitates [39]. Human Oxal1l was originally identified as it could functionally complement the deficiency of an OXA1 null budding yeast strain indicating a conservation of function. However, this function may depend on the amino acid sequence or assembly process of yeast subunits that may differ from human counterparts [40]. 

Once inserted into the IMM human Cox1 follows a similar assembly pathway described above as in *S. cerevisiae*. Cox1 assembles into the MITRAC complex, an assembly intermediate composed of homologues to *S. cerevisiae* assembly factors. This complex was initially characterised by isolation of MITRAC12 (COA3) to identify associated proteins which included Cox1, other COX subunits, Cox14 Cox15, Cox16, SURF1, MITRAC15 (Coa1), CMC1 and TIM21 [41]. TIM21 is a component of the TIM23 IMM translocase complex and may provide a control link between COX biogenesis and polypeptides imported from the cytosol. Further characterisation of the MITRAC complex identified a metazoan specific assembly factorMITRAC7that associated after structural subunits COX4i and COX6C. MITRAC7 abundance provided a control point for Cox1 incorporation into fully assembled COX. Decreased levels of MITRAC7 lead to the degradation of Cox1, while increased amounts resulted in sequestration of Cox1 and inhibition of COX formation [42]. The complexity of these assembly processes is demonstrated by cross-talk of assembly factors in more than one assembly pathway, for example, MITRAC15 has been shown to regulate translation of the Complex I subunit ND2 [42] 

**Figure 1:**
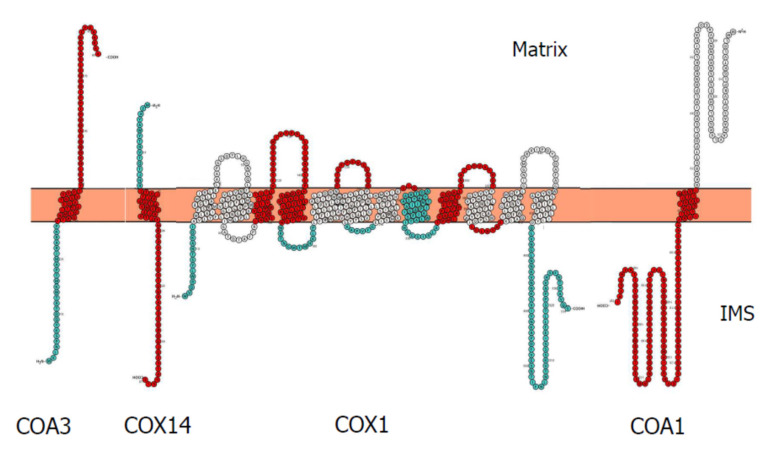
Representation of the topologies and isoelectric point of Cox1p, Cox14p, Coa1p and Coa3p. Domains coloured red and blue are >1 pH unit lower or higher than pI of the entire protein. IMS: inter-membrane space; COX: cytochrome *c* oxidase COA: Cytochrome *c* Oxidase Assembly Factor.

As the largest subunit of COX, Cox1p undergoes the most maturation steps and is also subject to various quality control checks as the mRNA is translated into the polypeptide that is inserted into the IMM. The Cox1p polypeptide contains amino acid sequences that vary in hydrophobicity, with those more hydrophobic residing in the IMM and connected to each other by hydrophilic stretches of amino acids. As with all multi-span integral membrane proteins some of the hydrophilic stretches need to be translocated from the matrix to the IMS through the hydrophobic IMM phospholipid bilayer which uses specific membrane localised translocation complexes. The first assembly factors that Cox1p initially associates with are Cox14p, Coa3p and Coa1p [28,43]. These are small proteins with a single trans-membrane spanning region with one of two membrane topologies. Biochemical fractionation and protease resistance determined that the amino termini of Cox14p and Coa1p are localized in the matrix and the carboxy-terminus is in the IMS while the amino-terminus of Coa3p is localized to the IMS and the carboxy-terminus is localized in the matrix [43,44,45,46]. These proteins all harbour canonical amino-terminal mitochondrial targeting sequences with a basic isoelectric point. Coa3p has an unexpected topology where the basic amino-terminus is exposed to the IMS [43]-perhaps indicating a more complex insertion process using matrix to IMS membrane insertion found in mitochondrial encoded genes mediated by Oxa1p. The three regions of these proteins in the matrix, IMM and IMS have different isoelectric points, perhaps indicating different functions (Table 2). Cox1p also has different regions that have different isoelectric points perhaps enabling interactions governed by domain charge (Figure 1). Complementary charges may assist the insertion of domains with a charge that may impact on efficiency of membrane insertion. Coa3p has a highly basic trans-membrane domain that could neutralise acidic regions of Cox1p in trans-membrane domains 3, 4 or 9 or IMS loop 5. Coa1p and Cox14p have acidic regions that could assist in translocation across the IMM such as the amino-terminus or loops 2, 3, or 4 or the carboxy terminus (Figure 1). 

The interaction between these three assembly factors and Cox1p has not been resolved to understand whether all three associate simultaneously or whether there is an ordered association. It can be speculated that these assembly factors are involved in insertion of Cox1p trans-membrane domains into the IMM or chaperone these domains as they are released from Oxa1p into the IMM through complementary amino acid side chain interactions. In the absence of COX14, COA1 or COA3, Cox1p tends to form aggregates in the IMM with Cox2p, Var1p or Cob1p that are not incorporated into functional COX [47]. Further investigation of these interactions at the amino acid level by mutagenesis will lead to understanding of the Cox1p-assembly factor interactions. COX14 was initially suggested to be a negative regulator of Cox1p translation as deletion allowed for expression/stabilisation of Cox1p when other assembly factors or structural subunits are absent [44]. Cox14p is part of an assembly intermediate composed of Coa3p, Mss51p and Ssc1p that acts as a regulator of Cox1p translation. Mss51p acts as a translational activator of COX1 mRNA. Mss51p remains associated with this complex if the downstream assembly process is interrupted by gene mutation or deletion or co-factor/prosthetic group absence. This ensures assembly is halted prior to association with other structural subunits that may result in defective and potentially harmful intermediates [43]. As an added layer of regulation, Mss51p also requires direct binding to haem to activate Cox1p translation [48]. This complex regulatory mechanism ensures sufficient abundance of prosthetic groups and assembly factors and structural subunits for complete COX assembly. 

**Figure 2:**
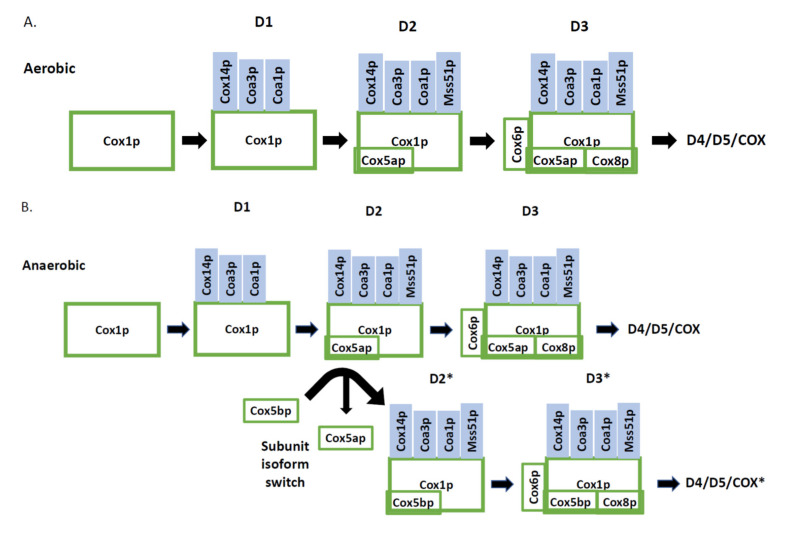

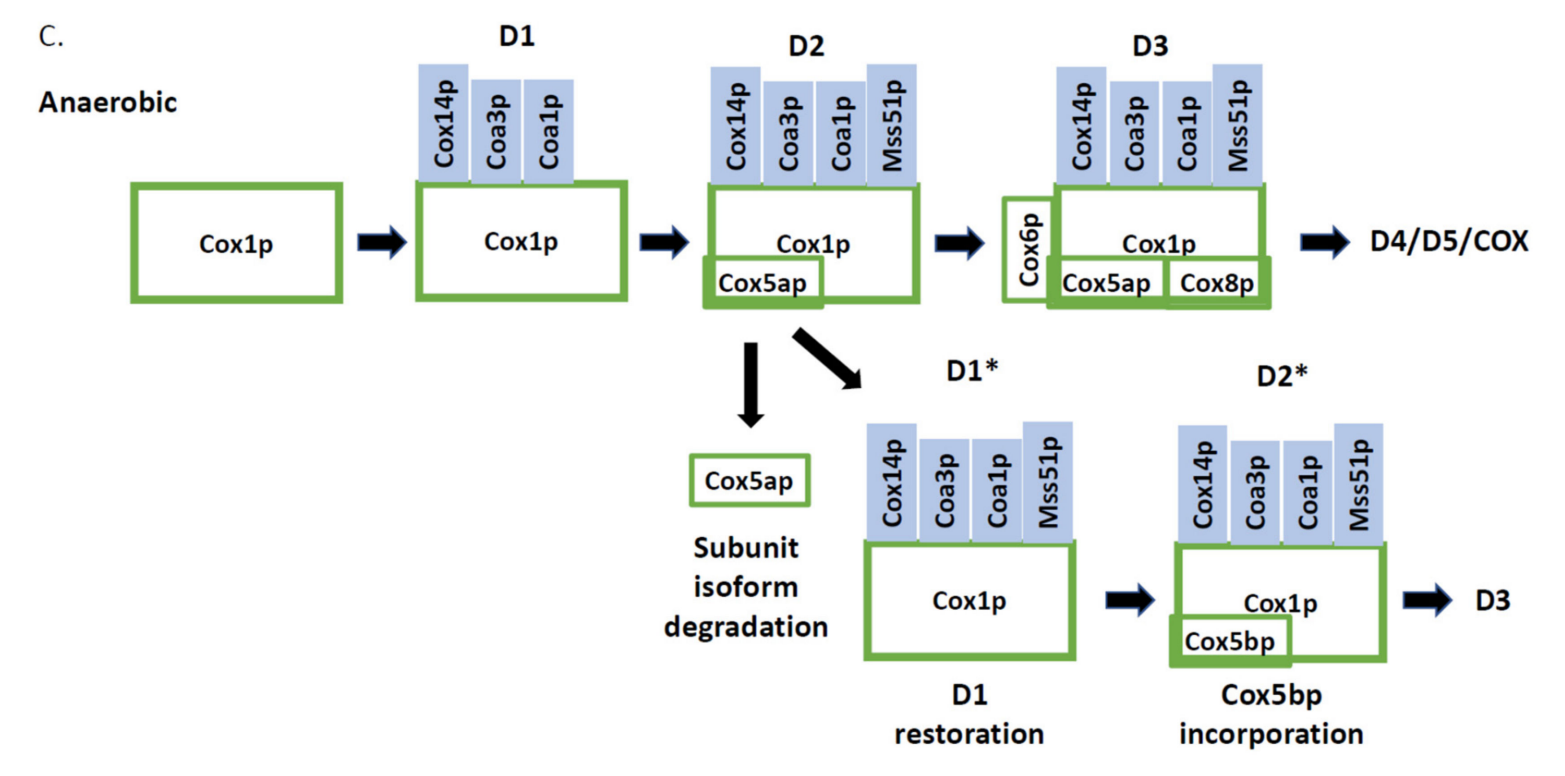
Hypothetical mechanisms for Cox5a/bp incorporation into COX during transition from aerobic to anaerobic growth conditions. (**A**) Cox1p assembly under aerobic conditions. (**B**) Subunit Cox5a/bp switch using a hypothetical subunit isoform switch mechanism. (**C**) Hypothetical subunit degradation mechanism where Cox5ap is degraded to reform the earlier assembly intermediate which can then accept newly expressed Cox5bp. D1, D2, D3, D4 and D5 represent discrete Cox1p containing assembly intermediates. The asterisks (*) represent hypothetical assembly intermediates with altered subunits from the earlier assembly intermediate not marked with the asterisk.

When Mss51p associates with the early Cox1p assembly intermediate the first structural subunit associates-in this case Cox5ap or Cox5bp [20]. The COX5A and COX5B isoforms are reciprocally expressed in aerobic and anaerobic conditions. The human orthologues of COX5A/B are COX4i/ii which are expressed in a similar way. The proposed reason for these isoforms is to allow for efficient transfer under different oxygen concentrations. Under low oxygen conditions in human cells there is an increase in ROS and the different isoforms allow for efficient transfer under these conditions [49]. Under a transition from aerobic to anaerobic conditions the mechanisms of the transition from one isoform to another differs in budding yeast and human. In humans the conserved mitochondrial protease Lon is responsible for degradation of the subunit to be removed, while expression of the other subunit is increased by enhanced transcription [49]. Human airways and placenta express COX4ii preferentially over COX4i indicating a specialised requirement in these tissues for COX activity. Expression in these tissues is due to the transcription factors CHCHD2, CXXC5 and RBPJ [50]. CHCHD2 and CXXC5 are not well characterised proteins, but RBPJ is a crucial transcriptional mediator in the Notch signalling pathway [51]. In budding yeast, COX5A/B are reciprocally expressed by oxygen concentration. Haem synthesis occurs only in aerobic conditions which activates the Hap2/3/4/5p transcriptional complex to express COX5A. Haem also activates Hap1p which activates the transcription repressor Rox1p-this results in repression of COX5B [52]. The kinetic pathway occurring to exchange subunits most likely depends on biogenesis of new COX rather than exchange of subunits; however, this has not been demonstrated experimentally. With Cox5 isoforms being the first structural subunits to associate with assembling Cox1p proposes the notion that this complex could enable subunit isoform exchange or subunit degradation followed by association of the other subunit (Figure 2). Regulation of COX activity in humans and budding yeast also involves the hypoxia regulated genes RCF and human homologues HIGD1A/HIGD2A that regulate COX assembly and incorporation of hypoxia sensitive subunits along with the formation of budding yeast and human supercomplexes [14]. 

The remaining assembly factor associating with Cox1p is Shy1p. This is a highly conserved gene found in prokaryotes and higher eukaryotic multicellular organisms demonstrating a fundamental process in the assembly process. Evidence supports Shy1 involvement in the incorporation of haem into the Cox1p assembly module along with Coa2p [46,53]. Shy1p interacts with the haem A synthase Cox15p supporting the role in haem A incorporation [54]. Shy1p exists in a Cox1p intermediate containing the structural subunits Cox6p and Cox8p [20]. A specific group on COX specific haem handling proteins are required for synthesis and modification of haem for incorporation into Cox1p. Cox10p farnesylates haem B to form haem and Cox15p modifies haem O to form haem A [55,56]. Pet117p is responsible for stabilising Cox15p in a functional oligomeric form [57]. The assembly pathway also involves the general mitochondrial chaperone Ssc1p and co-chaperone Mdj1p that may integrate the Cox1p specific assembly pathway to the general protein folding environment of the mitochondria [43,46]. 

Copper incorporation into Cox1p requires the function of a series of chaperones. Copper is delivered by Cox11p, an IMM localised protein that associates with the mitochondrial ribosome. Free cellular copper is eventually bound by Cox17p involving the homologues Cox19p and Cox23p in the IMM and IMS which transfers bound copper to Cox11p, which is then thought to add copper to Cox1p to form the copper B centre [58,59,60]. When COX11 or COX17 are absent COX cannot assemble as the action of the Mss51p-Cox1p complex stalls COX1 translation causing a decrease in Cox1p abundance. Similar to when other genes are deleted if COX14 is also deleted Cox1p translation is restored, however, this does not restore full COX assembly [44]. This highlights the important step of control of COX biogenesis at the point of Cox1p translation. The Cox17p homologue, Cox19p is proposed to protect Cox11p from oxidation and retain copper binding capability at redox sensitive cysteine residues [61]. With regards to the other Cox17p homologue, Cox23p absence can be suppressed by a specific mutation I101F mutation in Cox1p. This residue is located in the third trans-membrane domain; however, the function of the protein is unknown. Other copper chaperones are involved in Cox1 biogenesis such as Cmc1p and Cmc2p. These two proteins are required for COX assembly and function together to incorporate copper into COX; however, the exact role is not understood. Cmc1p and Cmc2p are conserved in higher mammals and result in deficiency and are also involved in correct copper incorporation into the copper-requiring enzyme superoxide dismutase-1 [62,63]. Human CMC1 is observed as part of early Cox1 intermediates containing Cox14 and Coa3. A metazoan specific assembly factor, MITRAC7, associates with the MITRAC complex, composed of human orthologues of Coa1/MITRAC12, Coa3/MITRAC5 and Cox14, along with structural subunits COX4i (yeast Cox5ap orthologue) and COX6C (yeast Cox9p orthologue) indicating function as a late stage Cox1 assembly factor [42].

Currently, the exact intermediates that incorporate haem or copper into the binuclear centre of Cox1p are not known. This has not been directly observed in purified intermediates which are low in abundance. There is also still a lack of resolution of the whole assembly process of Cox1p and whether proteins simultaneously associate or whether there are discrete steps that have not been resolved by current experimental techniques. 

### 3.2. Cytochrome c Oxidase Subunit 2 Assembly

Cox2p is a core COX subunit encoded by mitochondrial DNA with two trans-membrane spanning domains with a copper A site required for electron transfer. Cox2p requires a number of accessory membrane insertion proteins for correct topology in the IMM described above where both the amino and carboxy termini are located in the IMS. In humans, Cox20, the Cox2p membrane insertion chaperone, associates with TMEM177, an IMM protein with homologues in vertebrates [64]. Upon insertion into the IMM the copper co-factor is incorporated. There are several copper chaperones involved in this process including those involved in copper association in Cox1p. Copper is directed to Cox2p specific copper chaperones using Cox17p, a shared copper shuttle protein with Cox1p. Cox17p delivers copper to Sco1p, a specific Cox2p copper chaperone. Sco1p is an IMM localised protein that interacts directly with Cox17p and Cox2p, presumably delivering copper from Cox17p to Cox2p [65]. Sco1p has a close relative, Sco2p, that also contains the highly conserved copper binding domain found in many organisms that express COX [66]. Recently this process of Cox2p copper incorporation has been further characterised in human cell culture models. All of the components of this particular process are conserved and function in similar steps. More recently, COX16 investigations point to a function in copper delivery to Cox2 [67,68], but it is also identified in Cox1 containing complexes in budding yeast and humans potentially demonstrating a role for association between the assembly modules of Cox1 and Cox2 [30,68]. COA6 has been recently identified as a thiol-reductase for copper metallochaperones, especially for Sco1 and Sco2, to reduce disulphide bridges allowing required cysteine residues to remain reduced and interact with copper [69]. The assembly factors Pet100, Pet117 and MR-1S are thought to associate once the Cox1 and Cox2 module form an assembly intermediate. MR-1S is a vertebrate specific protein [70]. MR-1S is a short isoform of the myofibrillogenesis regulator (MR-1) that has roles in cellular proliferation [71]. 

### 3.3. Cytochrome c Oxidase Subunit 3 Assembly

Cox3 is a conserved mitochondrially encoded subunit with seven trans-membrane domains but does not contain any co-factor or prosthetic group. In budding yeast no assembly factors have been identified in the biogenesis of the Cox3 assembly module, only structural subunits that interact in the final assembled structure, Cox4p, Cox7p and Cox13p, along with Rcf1 which stabilises associations with complex III for supercomplex formation and regulate activity during different metabolic states [13,27]. This perhaps indicates that there is a co-ordination of assembly of complex III and IV to ensure a stoichiometric production of ETC complexes ensuring efficiency of electron transfer and minimising electron leak. Rcf1p and its homologue Rcf2p have been shown to regulate COX activity and associate with COX supercomplexes under respiratory growth conditions [72]. The lack of specific assembly factors seen in *S. cerevisiae* potentially means the structural subunits act as chaperones for Cox3 insertion in the membrane and remain as part of the assembled active enzyme COX. In humans the role of HIGD2A as an assembly factor of the Cox3 module has been proposed. HIG2DA associates with Cox3 and other structural subunits in assembly intermediates and is also found in COX containing supercomplexes [14]. In addition, HIGD2A depletion results in decreased stabilisation of Cox3 and depletion from mitochondrial supercomplexes composed of Complex I, III and IV [73]. HIGD1A, a paralogue of HIGD2A, appears to have more of a role in the assembly of COX and Complex III supercomplexes but has been found to associate with COX4i and COX5A [14] 

## 4. Mutations in Cytochrome *c* Oxidase Assembly Factors as Cause of Human Disease

Understanding of COX assembly has also been captured from the study of patients suffering from disease caused by mutations in these genes. Mutations in genes could result in the absence of assembled COX but also in mutation in critical residues for the activity of the enzyme. The inheritance pattern of these diseases also contributes to the severity of the disease. This is why mitochondrial diseases present as a heterogeneous spectrum of disorders that are difficult to diagnose and treat. A number of mutations in assembly factors are the cause of these diseases [11]. The most common cause of complex IV deficiency is through mutation of SURF1. There are at least 36 different mutations in this gene that result in disease, these are located throughout the length of the gene and result in missense and nonsense mutations. Some of these have been introduced into budding yeast SHY1 at conserved sites and result in the accumulation of intermediates at different stages of Cox1p module assembly. This indicates that Shy1p function can be sensed by the Mss51p checkpoint, but has functions later in the assembly process [74]. The wide variety of disease causing SURF1 mutations, along with the evidence of a weak and spontaneously suppressible respiratory defect in budding yeast, indicate that defects in SURF1 can be overcome by changes in a variety of other genes in the Cox1 assembly pathway. A similar explanation may be behind the number of mutations identified in the copper chaperones, COX10 and COX15 [75,76,77]. There are a number of homologues in this pathway that if altered in expression could explain the disease. Furthermore, as elevated copper can suppress yeast mutations, alterations in cellular copper handling could explain how a mutation is overcome to enable viability. Mutations in other genes are much less frequent and this is most likely explained by a defect that cannot be overcome by changes in gene expression. This also indicates that during embryonic development there is an essential function lacking when a mutation in other genes are present that cannot be overcome and the embryo is not viable. There are several examples where genes harbouring mutations lead to COX deficiency, but the role has not been fully characterised. CEP89 was identified as the mutated gene in a patient with COX deficiency. This mutation caused loss of COX activity and function [78]. The exact role of CEP89 in this process still has to be determined. In contrast to *S. cerevisiae*, a point mutation in COX14 results in decreased synthesis of COX1 with an expected loss of COX expression and activity potentially highlighting a divergence in the assembly process [79]. Mutations in COA3, which functions at a similar point to COX14, also result in a mitochondrial disease due to specific loss of COX expression [80]. Mutations in COA5, the human homologue of Pet191p, results in infantile cardioencephalomyopathy, caused by COX deficiency. Analysis of native complexes demonstrated a Cox1 containing assembly intermediate that may represent MITRAC [81]. COA7 mutants result in COX deficiency causing leukoencephalopathies and peripheral neuropathies [82,83] and this deficiency can be restored by inhibiting cytosolic degradation of COA7 indicating these mutations delay COA7 import into mitochondria which are still capable of contributing to COX assembly [84]. Mutations in PET100 also cause COX deficiency through a truncation and an import defect [85,86,87]. PET117 mutations cause COX deficiency, most likely through its role in assisting haem synthesis coupling to association into Cox1 [88]. In patients with COX10 mutations COX expression is decreased and Cox1 exists in a sub-assembly that migrates similar to MITRAC [89,90]. COX15 mutations also show similar deficiencies of COX; however, the assembly intermediates in these patients have not been investigated [56,77,91,92,93]. Mutations in the copper handling proteins, SCO1 and SCO2, have also been identified resulting in COX deficiency [94,95,96,97,98,99]. COA8, a less characterised protein identified as having a role in programmed cell death, when mutated can also cause COX deficiency [100].

## 5. Summary

In summary, the assembly of COX is a conserved process that requires the essential function of many assembly factors, somewhat unexpectedly more than the number of structural genes. This indicates the complexity and importance of the process. Without a functional COX enzyme, mitochondria are not able to produce ATP which has devastating consequences in the context of severely debilitating diseases that often lead to early death. Through further understanding of how COX assembly occurs better points of diagnostic and therapeutic intervention will be developed to improve the quality of life of patients suffering from these diseases, and families facing choices that cause anxiety and distress.

## Figures and Tables

**Table 1:** Assembly factors required for maturation of Saccharomyces cerevisiae and human COX. The name of the gene represents it either being conserved in both organisms, only found in budding yeast (gene name/_) or only found in human (_/gene name).

Mitochondrial Subunit	Transcription/mRNA Processing	Translation	Proteolytic Processing/Protease	Membrane Insertion	Copper Association	Haem Association	Chaperone	Unknown
COX1	Cox24	Mss51p/_ _/TACO1	Oma1	Oxa1 Mba1/_	Cox11 Cox17 Cox19 Cox23 Cmc1 Cmc2	Coa2 Cox10 Cox15 Shy1/SURF1	Coa1/MITRAC15 Coa3/MITRAC12 Cox14 Mdj1 Ssc1 _/MITRAC7	
COX2	–	Pet111	Cox20 Imp1 Imp2 Som1	Oxa1 Cox18 Pnt1 Mss2	Cox16 Cox17 Cox19 Cox23 Coa6 Cmc1 Cmc2 Sco1 Sco2 _/TMEM177	–	Cox20	
COX3	–	Pet54 Pet122 Pet494		–	–	–	Rcf1p/HIGD2A	
2 or more genes	LRPPRC	–		–	–	–	–	
Unknown								_/CEP89 _/COA7 _/COA8 Pet191/Coa5

**Table 2:** Calculated theoretical isoelectric points of matrix and IMS localised domains of Cox14p, Coa1p, and Coa3p using “ExPASy compute pI/MW” tool. Red and light blue indicate pI of domain in > 1 pH unit lower or higher than overall protein pI.

Protein	Matrix	IMS	Trans-Membrane	Total
Cox14p	9.69 N	4.58 C	5.52	7.51
Coa1p	10.94 N	5.66 C	5.52	10.12
Coa3p	5.48 C	11.07 N	8.43	9.83
